# Validation of Reliable Reference Genes for Real-Time PCR in Human Umbilical Vein Endothelial Cells on Substrates with Different Stiffness

**DOI:** 10.1371/journal.pone.0067360

**Published:** 2013-06-28

**Authors:** Gan Chen, Lian Zhao, Jiantao Feng, Guoxing You, Quanmei Sun, Penglong Li, Dong Han, Hong Zhou

**Affiliations:** 1 Institute of Transfusion Medicine, Academy of Military Medical Sciences, Beijing, China; 2 National Center for Nanoscience and Technology, Beijing, China; Scuola Superiore Sant'Anna, Italy

## Abstract

**Background:**

The mechanical properties of cellular microenvironments play important roles in regulating cellular functions. Studies of the molecular response of endothelial cells to alterations in substrate stiffness could shed new light on the development of cardiovascular disease. Quantitative real-time PCR is a current technique that is widely used in gene expression assessment, and its accuracy is highly dependent upon the selection of appropriate reference genes for gene expression normalization. This study aimed to evaluate and identify optimal reference genes for use in studies of the response of endothelial cells to alterations in substrate stiffness.

**Methodology/Principal Findings:**

Four algorithms, GeNorm^PLUS^, NormFinder, BestKeeper, and the Comparative ΔCt method, were employed to evaluate the expression of nine candidate genes. We observed that the stability of potential reference genes varied significantly in human umbilical vein endothelial cells on substrates with different stiffness. B2M, HPRT-1, and YWHAZ are suitable for normalization in this experimental setting. Meanwhile, we normalized the expression of YAP and CTGF using various reference genes and demonstrated that the relative quantification varied according to the reference genes.

**Conclusion/Significance:**

Consequently, our data show for the first time that B2M, HPRT-1, and YWHAZ are a set of stably expressed reference genes for accurate gene expression normalization in studies exploring the effect of subendothelial matrix stiffening on endothelial cell function. We furthermore caution against the use of GAPDH and ACTB for gene expression normalization in this experimental setting because of the low expression stability in this study.

## Introduction

Change in the mechanical property of cells and their microenvironments and the relevance to biological functions have recently attracted increased attention. For example, mechanical microenvironments may regulate cellular functions relevant to development, homeostasis, and disease [1]–[3]. Many mainly pathological conditions, including aortic stiffness and liver fibrosis, result in significant mechanical changes at the whole organ, regional, and cellular levels [3]–[5]. Therefore, we need to determine the genetic and molecular basis of the mechanical changes and then identify these biomolecules and their signaling pathways for the development of future drug treatments.

Among the widely used methods, real-time quantitative reverse transcription polymerase chain reaction (qRT-PCR) is a rapid and sensitive method for measuring gene expression. Despite being a very powerful technique to accurately quantify gene expression, several determinants, such as input sample, RNA extraction, efficiency of reverse transcription from RNA to complementary DNA and PCR efficiency, should be taken into account. Therefore, normalization of qRT-PCR data with suitable internal reference genes (RGs) is required [6]. The ideal RG should be expressed at a constant level between samples or under different conditions. Nevertheless, there is now increasing evidence suggesting that the expression of RGs often varies significantly under different experimental conditions. Thus, identification of reliable RGs is a prerequisite for qRT-PCR experiments.

In our present study, we investigate reliable reference genes for real-time PCR in human umbilical vein endothelial cells (HUVECs) on substrates with different stiffness. It is well established that the subendothelial layers of blood vessels become stiff in cardiovascular diseases [2], [7]. The mechanical changes, therefore, may lead to dysregulate the endothelial layer and influence disease states [8], [9]. Evidence suggests that proper vessel compliance is critical for endothelial response to hemodynamic forces. Peng et al. showed that when ECs are plated on stiff-walled tubes and subjected to physiological pressures, endothelial NO synthase expression decreased in comparison to production by cells on distensible tubes [10]. Consequently, a better understanding of the response of endothelial cells to alterations in substrate stiffness could shed new light on the development of cardiovascular disease.

Previous studies demonstrated the importance of substrate stiffness on the gene expression of various cell types [11]–[18], such as Yes-associated protein (YAP) and connective tissue growth factor (CTGF) (Table 1). Endothelial cells responding to subendothelial matrix stiffening is a process of cells undergoing numerous morphological changes [19], [20], which are accompanied by substantial changes in biochemical and metabolic processes and alteration in structural proteins [10], [21]. Significant modifications in gene expression, including the widely used RGs, contribute to these cellular changes. To date, few studies dealing with how cellular gene expression responds to alterations in substrate stiffness reported on the stability of RGs prior to use in the studies. Validation of stably expressed RGs for endothelial cells on a variety of substrate stiffness is especially important.

**Table 1:** Some examples of genes that exhibit different expression levels as a function of substrate stiffness.

Gene name	Cell lines	Ref
cyclin A, p27, and Rb	Human umbilical vein endothelial cells	[11]
IL-2	Primary peripheral blood lymphocytes	[12]
TNF-α, IL-1β, and IL-6	Murine bone marrow-derived primary macrophages	[13]
α-SMA, ET-1, and IL-1β	Colonic human myofibroblast cells	[14]
YAP, TAZ, TGM2, and sFRP-1,	Primary human trabecular meshwork cells	[15]
α-SMA and CTGF,	Primary valvular interstitial cells	[16]
COX-2, PGE2, MMP10, and MMP3	Lung fibroblasts	[17]
Actin, Tubulin, and PFKP-1	A549	[18]

The aim of this study was to identify and evaluate the expression stability of nine candidate RGs in a widely used *in vitro* model of the HUVECs in order to explore the effect of subendothelial matrix stiffening on endothelial cell function. Polyacrylamide gels were prepared to obtain three different stiffness values that mimic pathophysiological states (table 2), based on the currently published range of vascular basement membrane compliances of 2.5–8 kPa [22]–[24].

**Table 2:** Characterization of the polyacrylamide gels.

	Acrylamide %	Bisacrylamide%	Measuring elastic modulus (kPa)
**Soft**	3	0.1	0.91±0.73
**Medium**	5	0.15	2.08±1.32 #
**Stiff**	8	0.48	12.39±7.85 &*

Data are mean±SD from three independent experiments. Means were compared by ANOVA followed by Student-Newman-Keuls multiple range text. #*P*<0.05, Medium vs. Soft. *P<0.05, Stiff vs. Soft. &*P*<0.05, Stiff vs. Medium.

## Results

### Selection of Candidate RGs and Amplification Specificity

Nine candidate RGs were selected for this study. These candidates are widely used and recognized RGs, which have been described in the literature and represent several functional classes to minimize the possibility of co-regulation (Table 3).

**Table 3:** Symbols, names, accession numbers and functions of the candidate RGs evaluated.

Gene symbol	Gene name	Accession number	Function
**ACTB**	Beta -actin	NM_001101	Cytoskeletal structural protein
**GAPDH**	Glyceraldehyde-3-phosphate dehydrogenase	NM_002046	Carbohydrate metabolism
**HPRT1**	Hypoxanthine phosphoribosyl-transferase 1	NM_000194	Purine synthesis through the purine salvage pathway
**YWHAZ**	Tyrosine 3-monooxygenase/tryptophan 5–monooxygenase activation protein, zeta polypeptide	NM_003406	Protein domain in specific binding
**TBP**	TATA box binding protein	NM_003194	Transcription initiation from RNA polymerase II promotor
**G6PD**	Glucose-6-phosphate dehydrogenase	NM_000402	Involved in the normal processing of carbohydrates
**RPL13A**	Ribosomal protein large L13a	NM_012423	Structural constituent of ribosome
**18S**	RNA,18S ribosomal 1	NR_003286	Cytosolic small ribosomal subunit, translation
**B2M**	Beta-2-microglobulin	NM_004048	Beta–chain of MHC class I molecules

Their respective PCR amplification efficiencies were calculated as the first step. Table 4 lists the amplification efficiency for each of the candidate RGs that ranged from 94% to 111%. The performance of each amplification primer set was tested by qRT-PCR. Melting curve analysis confirmed the presence of a single PCR product from all samples with no primer-dimers. The amplifications were also confirmed by the presence of a single band of the expected size for each primer pair in 2% agarose gel electrophoresis (Figure S1).

**Table 4:** Primer Information for the selected candidate reference genes.

Gene symbol	Forward primer	Reverse primer	Amplicon size (bp)	PCR efficiency(%)
**ACTB**	CATCGAGCACGGCATCGTCA	TAGCACAGCCTGGATAGCAAC	211	94.2
**GAPDH**	GTCAGCCGCATCTTCTTTTG	GCGCCCAATACGACCAAATC	100	108.3
**HPRT1**	GACCAGTCAACAGGGGACAT	AACACTTCGTGGGGTCCTTTTC	195	103.7
**YWHAZ**	ACTTTTGGTACATTGTGGCTTCAA	CCGCCAGGACAAACCAGTAT	94	103.8
**TBP**	GAGCTGTGATGTGAAGTTTCC	TCTGGGTTTGATCATTCTGTAG	118	107.8
**G6PD**	CCGTCACCAAGAACATTCACG	GGACAGCCGGTCAGAGCTCT	107	103.1
**RPL13A**	CCTGGAGGAGAAGAGGAAAGAGA	TTGAGGACCTCTGTGTATTTGTCAA	126	99.3
**18S**	CAGCCACCCGAGATTGAGCA	TAGTAGCGACGGGCGGTGTG	252	99.7
**B2M**	CACCCCCACTGAAAAAGATGAG	CCTCCATGATGCTGCTTACATG	106	110.9

### Expression Levels of Candidate RGs

**Figure 1:**
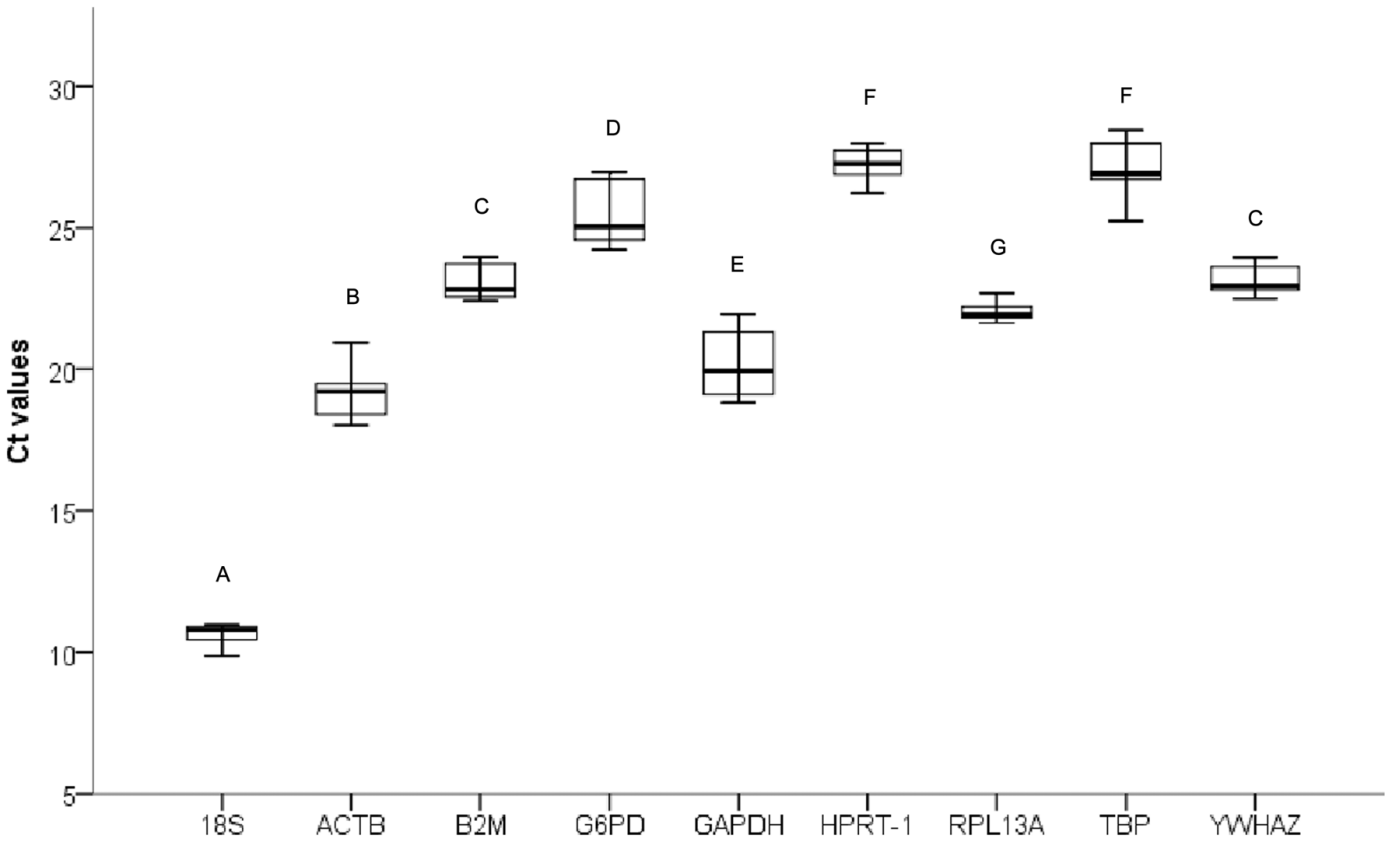
Distribution of qRT-PCR quantification cycle values for the candidate RGs. Box plot graphs of Ct values for each RG tested in all samples. Vertical lines indicate range of values, and the median Ct value is represented as black horizontal line within the box plot. The box indicates the 25th and 75th percentiles. Letters indicate a significant difference in average Ct value. Average Ct values that have the same letter are not significantly different (*P*>0.05).

The expression levels of all nine candidate RGs were evaluated as threshold cycle (Ct) values with three biological and three technical replicates. The box plot of the Ct values of all candidate RGs show the differences in transcript levels between RGs (Figure 1). The expression levels of these RGs varied widely, with Ct values ranging from 9.87 to 28.44 cycles. 18S was the most abundantly transcribed gene, with a mean Ct value of 10.62 cycles, whereas HPRT-1 showed the lowest level of expression in all samples, with a mean Ct value of 27.21 cycles. The individual reference genes had different expression ranges across samples. Among the nine candidate RGs in this study, ACTB and GAPDH had large expression variations in their transcript levels (2.92 and 3.11 cycles, respectively), while RPL13A, 18S, and YWHAZ had much lower expression variation (1.05, 1.1, and 1.27 cycles, respectively). The wide expression range of the candidate RGs indicated the importance of accurately calculating the RGs levels with the widely used statistical algorithms described below.

### Analysis of Candidate RGs Stability

Four distinct algorithms, GeNorm^PLUS^, Normfinder, Bestkeeper and the Comparative ΔCt method, were employed to further evaluate the expression stability of the candidate RGs individually.

### GeNorm^PLUS^ Analysis

The GeNorm algorithm applies a statistical algorithm to calculate the average stability measure (M). A lower value of average expression stability M indicated more stable expression [6]. The Pairwise variation (V) parameter was calculated to determine the optimal number of RGs required for normalizing the expression of genes of interest. Generally, 0.15 was used as a cutoff value to determine the optimal number of RGs [6]. Below this value, the addition of extra RGs does not improve the expression stability of the RG set and is therefore not recommended.

**Figure 2:**
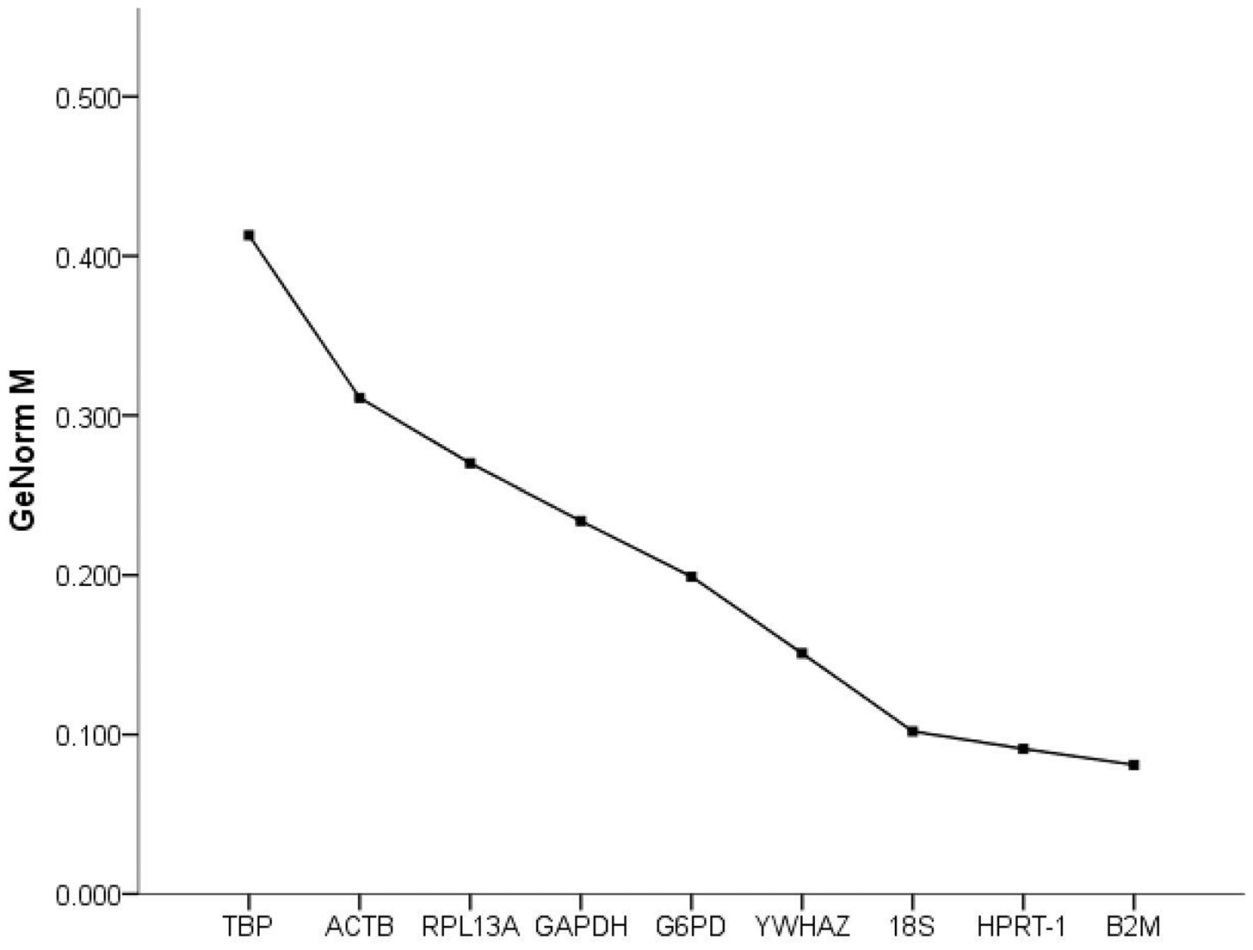
Expression stability values (M) of candidate RGs calculated by GeNorm^PLUS^. M values of the remaining candidate citrus RGs during stepwise exclusion of the least stable citrus RG in the different subsets. A lower average M value indicates more stable expression.

**Figure 3:**
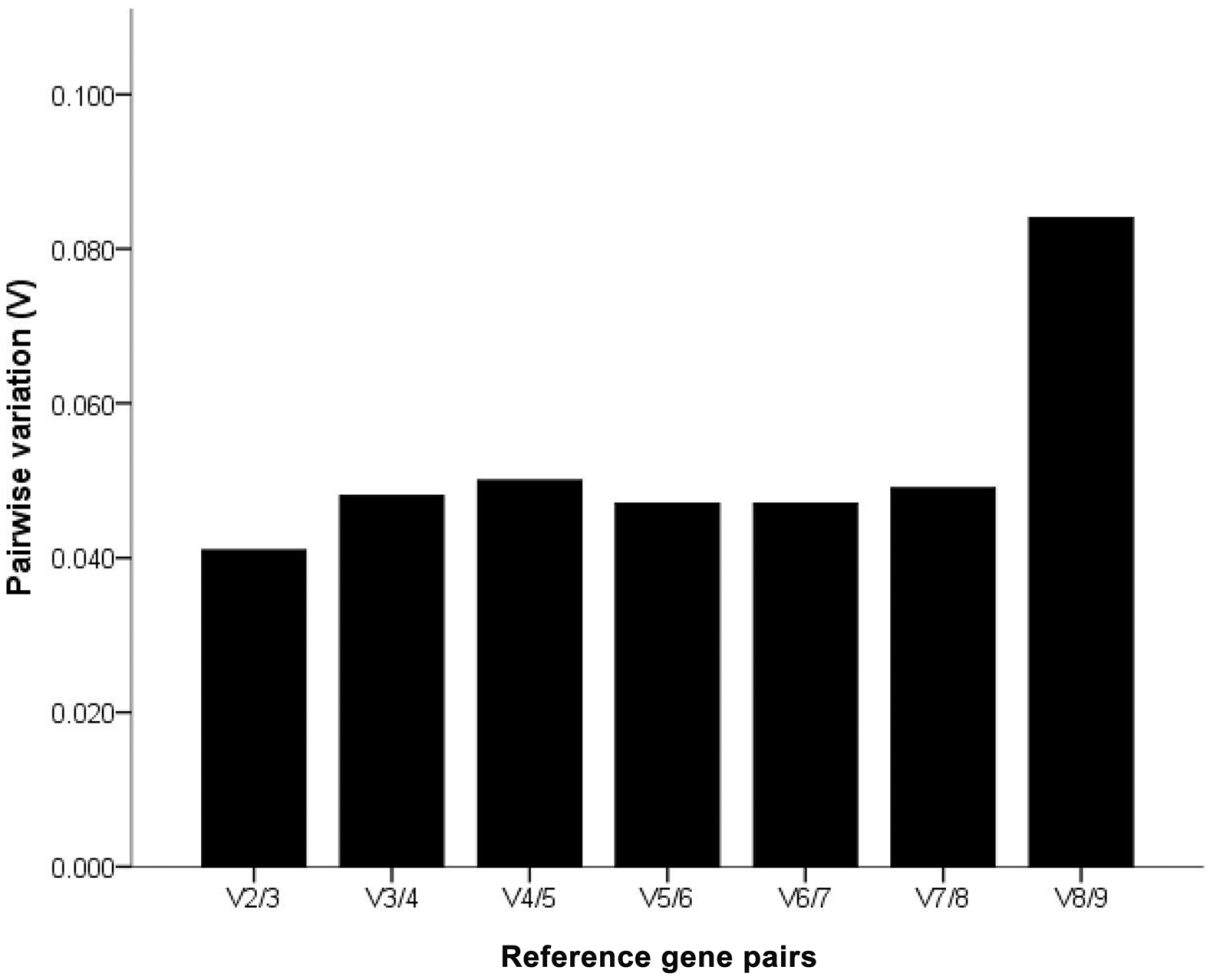
Determination of the optimal number of RGs for qRT-PCR normalization by GeNorm^PLUS^. The pairwise variation (V) calculated using GeNorm^PLUS^ to determine the optimal number of RGs for accurate qRT-PCR normalization in different experimental settings. V2/3 exhibited the value below the cut-off value of 0.15, indicating that use of 2 RGs for normalization is necessary, whereas addition of a third RG is optional.

All candidates were ranked based on M values (Figure 2). The nine selected candidate genes all reached the high expression stability criterion, with M<0.42, which is well below the default limit of 1.5 suggested by GeNorm^PLUS^. Of the candidates, the B2M gene had the lowest M value, followed by HPRT-1. Interestingly, ACTB, although frequently used for gene expression, had the lowest expression stability in this study. Moreover, the V parameter calculated by GeNorm^PLUS^ recommended the use of two RGs for reliable normalization in this experimental setting (Figure 3), and the addition of a third gene is optional.

### NormFinder Analysis

NormFinder is another algorithm used to determine the optimal RGs for qRT-PCR normalization. This algorithm accounts for intra- and intergroup variation in the normalization factor calculations and calculates a stability value (S) for the RGs. The lowest S value indicates the most stable RG expression.

According to NormFinder analysis, the best ranked RG was B2M, followed by HPRT-1, YWHAZ, and RPL13A (Table 5). TBP and GAPDH were the least stable RGs in the present study.

**Table 5:** Gene stability (S) values calculated by Normfinder.

Rank	Gene symbol	Stability Value
**1**	B2M	0.122
**2**	HPRT-1	0.180
**3**	YWHAZ	0.226
**4**	RPL13A	0.468
**5**	ACTB	0.504
**6**	G6PD	0.532
**7**	18S	0.592
**8**	TBP	0.621
**9**	GAPDH	0.656

### BestKeeper Analysis

BestKeeper calculates the gene expression variation for candidate genes based on standard deviation (SD), coefficient of correlation (r) and percentage covariance (CV). The lowest SD value indicates the most stable RG expression.

As shown in Table 6, the BestKeeper analysis highlighted RPL13A and 18S as the most stable genes with the lowest SD (0.22 and 0.29, respectively), followed by YWHAZ, HPRT-1, B2M, ACTB, TBP, G6PD and GAPDH. The differences between the GeNorm^PLUS^ and BestKeeper results were expected because their statistical algorithms were distinct.

**Table 6:** Expression stability evaluated by BestKeeper.

Candidate Genes	ACTB	GAPDH	HPRT-1	YWHAZ	TBP	G6PD	RPL13A	18S	B2M
n	27	27	27	27	27	27	27	27	27
**geo Mean [Ct]**	19.04	20.01	27.21	23.30	27.08	25.42	22.01	10.62	23.07
**ar Mean [Ct]**	19.06	20.03	27.21	23.30	27.09	25.44	22.01	10.63	23.08
**Min [Ct]**	18.01	18.83	26.23	22.67	25.25	24.23	21.63	9.87	22.40
**Max [Ct]**	20.93	21.94	27.98	23.94	28.44	26.97	22.68	10.97	23.97
**std dev [+/− Ct]**	0.60	0.93	0.44	0.41	0.75	0.91	0.22	0.29	0.52
**CV [% Ct]**	3.17	4.64	1.62	1.76	2.76	3.58	0.98	2.74	2.27

### Comparative ΔCt Method Analysis

The Comparative ΔCt method assesses the most stable RGs by comparing the relative expression of “pairs of genes” within each tissue sample or each treatment [25]. This algorithm highlighted B2M and HPRT-1 as the most stable genes, followed by YWHAZ and RPL13A (Table 7); these findings are highly consistent with the GeNorm^PLUS^ and Normfinder results.

**Table 7:** Ranking of RGs stability.

Rank	GeNorm	NormFinder	BestKeeper	ΔCt method	Final ranking
**1**	B2M	B2M	RPL13A	B2M	B2M
**2**	HPRT-1	HPRT-1	18S	HPRT-1	HPRT-1
**3**	18S	YWHAZ	YWHAZ	YWHAZ	YWHAZ
**4**	YWHAZ	RPL13A	HPRT-1	RPL13A	RPL13A
**5**	G6PD	ACTB	B2M	G6PD	18S
**6**	GAPDH	G6PD	ACTB	ACTB	G6PD
**7**	RPL13A	18S	TBP	18S	ACTB
**8**	ACTB	TBP	G6PD	GAPDH	GAPDH
**9**	TBP	GAPDH	GAPDH	TBP	TBP

### Final Ranking of Candidate Reference Genes

RG rankings obtained with all four algorithms (GeNorm^PLUS^, Normfinder, BestKeeper, and the Comparative ΔCt method) were compared (Table 7). While RG rankings vary slightly by algorithm, a method previously described [26] was used to give an overall ranking of the best candidate RGs. The geometric means of the four ranking numbers were calculated, and the gene with a smaller geometric mean is the most stable RG. The recommended comprehensive rankings were also given in Table 7. Using the results from all four algorithms, an overall ranking of candidate RGs was obtained. B2M, HPRT-1, and YWHAZ represent the most reliable RGs in this experimental setting. The conventional RGs, GAPDH and ACTB were found to be less reliable and are not the good choices for RGs in this experimental setting.

### Validation of Reference Genes

**Figure 4:**
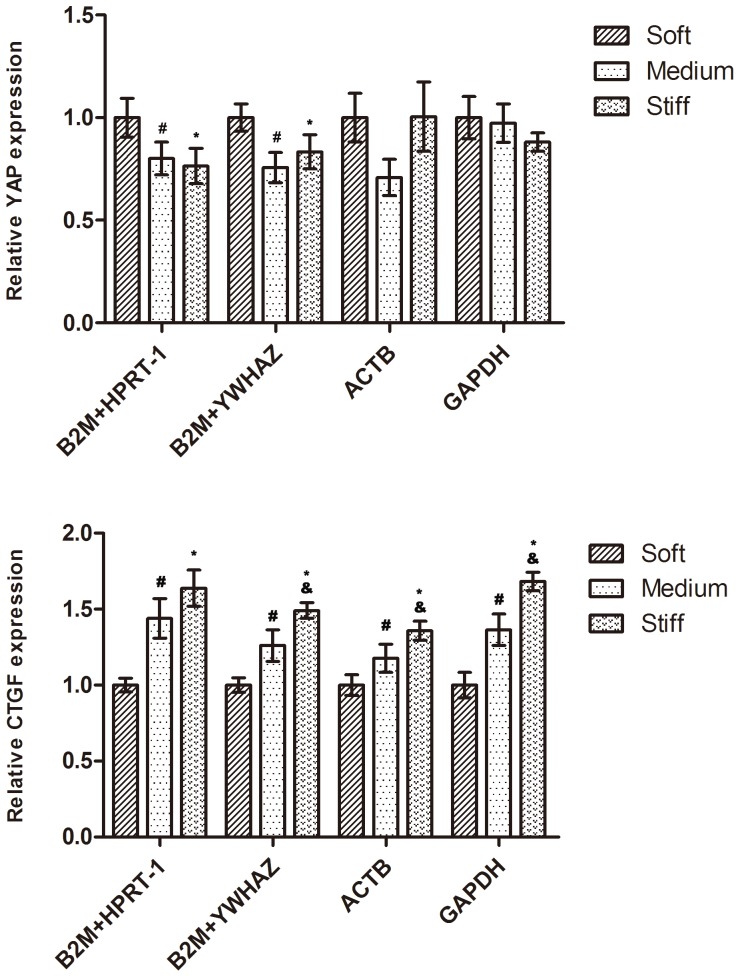
Expression levels of YAP and CTGF in endothelial cells on a variety of substrate stiffness. Genes were normalized to individual and/or combined RGs. Results are mean±SD, n = 3; Means were compared by ANOVA followed by Student-Newman-Keuls text. #*P*<0.05, Medium vs. Soft. *P<0.05, Stiff vs. Soft. &*P*<0.05, Stiff vs. Medium.

To show the effect of a reference gene on the outcome of a practical experiment, we evaluated the expression patterns of two genes, YAP and CTGF, using different normalization strategies. In previous studies, the transcription level of YAP was downregulated on stiffer substrates (18S as the RG) [15], and CTGF was significantly upregulated (18S as the RG) [16]. The 2 representative least stable RGs (ACTB and GAPDH) and a combination of 2 of the 3 most stable RGs (B2M, HPRT-1, and YWHAZ) were used as RGs for expression normalization (Figure 4). When B2M and HPRT-1 or YWHAZ were used as RGs, the expression of YAP decreased with substrate stiffness. Whereas when the least stable reference genes, ACTB and GAPDH, were used for normalization, YAP exhibited a similar expression level between different substrate stiffness. The CTGF exhibited a similar expression pattern between different normalization strategies. In addtion, we also evaluated the expression patterns of two functional genes, PAI-1 and tPA, using different normalization strategies (Figure S2). Both of these genes are known to play a crucial role in EC-mediated fibrinolytic activity. When B2M and YWHAZ or HPRT-1 were used as RGs, the expression of PAI-1 decreased with substrate stiffness. Whereas when the least stable reference genes, ACTB and GAPDH, were used for normalization, PAI-1 showed a different result when compared to the stable reference genes for normalization. Thus, these results reinforce the importance of validating reference genes prior to experimental applications.

## Discussion

Analysis of gene expression using qRT-PCR, which is a powerful method that combines high specificity and sensitivity, is a commonly used technology for gene expression analysis in response to different environmental conditions [27]. However, numerous studies have demonstrated that the performance of this technique is strongly dependent on a normalization strategy through the selection of appropriate RGs [28]–[30]. Thus, it is advisable to validate the expression stability of candidate RGs under specific experimental settings prior to use in qRT-PCR normalization. In this study, we sought to identify the appropriate RGs for normalizing qRT-PCR gene expression data in HUVECs responding to subendothelial matrix stiffening *in vitro.* Of nine candidate RGs, we identified B2M, HPRT-1, and YWHAZ as the most suitable RGs, using four widely recognized algorithms (GeNorm^PLUS^, Normfinder, BestKeeper, and the Comparative ΔCt method). We further found that both ACTB and GAPDH, which are frequently used for gene expression normalization in many experiments, were not suitable normalization controls in this experimental setting. The results from GeNorm^PLUS^, NormFinder, and the Comparative ΔCt method assessment were more consistent with each other than with the BestKeeper method.

The three most stably expressed genes in our experimental setting, B2M, HPRT-1, and YWHAZ, encode proteins with independent physiological functions. The protein encoded by B2M is a component of the major histocompatibility complex (MHC) class I [31]. HPRT-1 encodes an enzyme that plays a central role in the generation of purine nucleotides through the purine salvage pathway [32]. YWHAZ encodes the protein that mediates signal transduction by binding to phosphoserine-containing proteins [33]. Thus, these genes represent several distinct functional classes so as to minimize the possibility of co-regulation when combining these genes as RGs for qRT-PCR normalization in future studies.

Numerous morphological changes, which are accompanied by substantial changes in biochemical processes and metabolism and alteration in structural proteins, occur when cells adapt to substrates with different stiffness. Substrate stiffness regulates actin organization, cellular metabolism and protein synthesis in cells [21], [34], [35]. Byfield et al. showed that endothelial cells in stiffer gels exhibited more pronounced stress fibers and 1.5-fold greater actin staining [21]. Tilghman et al. demonstrated several of the proteins involved in the metabolic state are sensitive to changes in stiffness in cancer cells by measuring the rates of protein synthesis [36]. Thus, it is not that surprising to find that both ACTB and GAPDH are not suitable qRT-PCR normalization controls for these experiments. GAPDH and G6PD encode the key enzymes in the glycolytic pathway [37], [38], and ACTB encodes β-actin, which is involved in cell motility, structure and integrity.

Due to the original intention of our research, it is impossible in this study to research all human cell lines, and thus, these conclusions should be corroborated prior to application in other human cell lines. Furthermore, optimization of reference genes for real-time PCR associated with mechanical environments allows us to conduct more standardized biomechanopharmacology studies [39] in various patho/physiological stations.

In conclusion, we validated a stably expressed RG set for use in endothelial cells on a variety of substrate stiffness. B2M, HPRT-1, and YWHAZ were identified as the most stable RGs. Indeed, a combination of 2 genes out of these 3 genes is sufficient to provide accurate qRT-PCR normalization. Our results also demonstrated that special attention must be given to the choice of suitable RGs during the studies of the cell behaviors responding to the mechanical changes of their microenvironments.

## Materials and Methods

### Polyacrylamide Gel Preparation and Characterization

Polyacrylamide gels were prepared as described previously [40], [41]. Briefly, glass coverslips were treated with 3-aminopropyltrimethoxysilane and 0.5% glutaraldehyde after plasma cleaning (Harrick Plasma, Ithaca, NY). The PA gel premix solutions of acrylamide and bis-acrylamide (Bio-Rad, Hercules, CA) were mixed with tetramethylethylenediamene and ammonium persulfate, and the polymerizing solution was immediately added onto chloro-silanated glass slide after placing the glass coverslips on a drop of solution. The gels were allowed to polymerize at room temperature to a thickness of approximately 500 µm. Gel stiffness was manipulated by varying the acrylamide and bis-acrylamide ratio. Next, the gels were functionalized by UV irradiation of Sulfo-SANPAH in 50 mM HEPES buffer (pH 8.5) and then coated with 0.1 mg/ml type I collagen overnight at 4°C.

Force curves were acquired with an atomic force microscope (AFM, Agilent Technologies, CA). Silicon nitride cantilever (spring constant, 0.031 N/m; NT-MDT) with a spherical tip, of which the diameter was 16.46 µm, was used for measurements of Soft and Medium PA gels. For Stiff PA gels measurement, the silicon nitride cantilever (spring constant, 0.292 N/m; NT-MDT) with a borosilicate sphere of 11.9 µm in diameter was used. Force curves were obtained on at least seven different locations of each sample and twenty force curves were collected at each sample. Each force curve was taken at a rate of 1 Hz.

The force curves were analyzed using the Hertz model for a sphere in contact with a flat surface. In this model, the Young’s modulus E are calculated according to the following equation [42]:



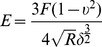
.Where *F* is the loading force, 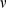 is Poisson’s ratio (assumed to be 0.3), 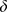 is the indentation depth (50 nm), *E* is the Young’s modulus, *R* is the radius of the silica sphere. Loading force was calculated by using Hook’s law, 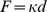, where 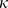 is the spring constant of the cantilever and *d* is the cantilever deflection. The characterization of the substrates is shown in table 2.

### Cell Culture and Treatments

Primary HUVECs were obtained from ScienCell Research Laboratories (ScienCell, Carlsbad, CA) and cultured on plastic flasks coated with 1% gelatin in endothelial cell growth medium-2 (EGM-2) supplemented with 2% FCS and growth factors (Lonza, Walkersville, MD) at 37°C under 5% CO_2_. Cells between passages 2 and 6 were used for all experiments. The cells were plated onto the previously prepared PA gels and grown for 48 hours. The medium was replaced once after 24 h.

### RNA Isolation and cDNA Synthesis

Total RNA was extracted using the RNeasy mini kit (Qiagen, Valencia, CA), according to the manufacturer’s instructions. The quantity and quality of RNA samples were determined using a Helios beta spectrometer (Thermo Scientific, Milford, MA). RNA samples with 260/280 ratio from 1.9 to 2.1 were used for further analysis.

First strand cDNA was synthesized by reverse transcribing 500 ng of total RNA with the RevertAid™ first strand cDNA synthesis kit (Fermentas life sciences, Vilnius, Lithuania) in a 20 µl reaction using random primers according to manufacturer’s instructions. The RT reaction sequence consisted of incubation at 25°C for 5 minutes, followed by 60 minutes at 42°C. The reaction was terminated by heating to 70°C for 5 minutes. The cDNA was stored at −20°C until the PCR experiments.

### Quantitative Real-time PCR Procedure

The qRT-PCRs were performed in 96-well plates with an ABI StepOne Plus Sequence Detection System (Applied Biosystems, Foster City, CA) using the SYBR® Green Realtime PCR Master Mix (Toyobo, Osaka, Japan). Thermocycling was performed using the following conditions: 94°C for 1 minutes, followed by 40 cycles of 94°C for 20 s, 59°C for 20 s, and 72°C for 25 s. The Ct values were automatically calculated using commercial software (StepOne Software V2.1, Applied Biosystems). The details of primer sequences are given in Table 4 and Table S1.

After completion of standard qRT-PCR, melting curve analysis demonstrated a single PCR amplicon for each reaction. The PCR efficiency was evaluated by the dilution series method using a mix of sample cDNAs as the template. A standard curve was generated using linear regression based on the threshold cycle (Ct) values for all dilution points in a series. The correlation coefficients (R^2^) and slope values were obtained from the standard curve, and the corresponding PCR amplification efficiencies (E) were calculated using the slope of the calibration curve according to the following equation: 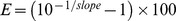.

### Determination of RG Expression Stability and Minimum Number of RGs Required

To assess the stability of candidate RGs, four widely recognized RG normalization algorithms were used, GeNorm^PLUS^ [6], NormFinder [43], BestKeeper [44], and the Comparative ΔCt method [25]. Four different algorithms were applied to data on three different stiffness and the same RGs were detected in all three different conditions.

### Statistical Analysis

Data are expressed as mean±standard deviation (SD) and analyzed using the Statistical Analysis System (SAS) software. Means of different groups were compared using one way analysis of variance followed by Student-Newman-Keuls text. *P* values <0.05 were considered significant.

## Supporting Information

Figure S1 **Specificity of qRT-PCR amplification.** (a) Amplified fragments were separated by 2% agarose gel. BM2000 represented DNA size marker. The fifth marker band corresponds to 250bp, and the last marker band corresponds to 100bp. (b) Dissociation curves of the nine amplicons showing single peaks.
(TIF)

Figure S2 **Expression levels of PAI-1 and tPA in endothelial cells on a variety of substrate stiffness.** Genes were normalized to individual and/or combined RGs. Results are mean±SD, n = 3; Means were compared by ANOVA followed by Student-Newman-Keuls text. #*P*<0.05, Medium vs. Soft. *P<0.05, Stiff vs. Soft. &*P*<0.05, Stiff vs. Medium.
(TIF)

Table S1 **Primer Information for YAP, CTGF, tPA, and PAI-1.**
(DOC)
