# Supplementary figures and images for: Validation of Reliable Reference Genes for Real-Time PCR in Human Umbilical Vein Endothelial Cells on Substrates with Different Stiffness
Source: PLoS One. 2013 Jun 28;8(6):e67360. doi: 10.1371/journal.pone.0067360 (PMC3696109; doi:10.1371/journal.pone.0067360)

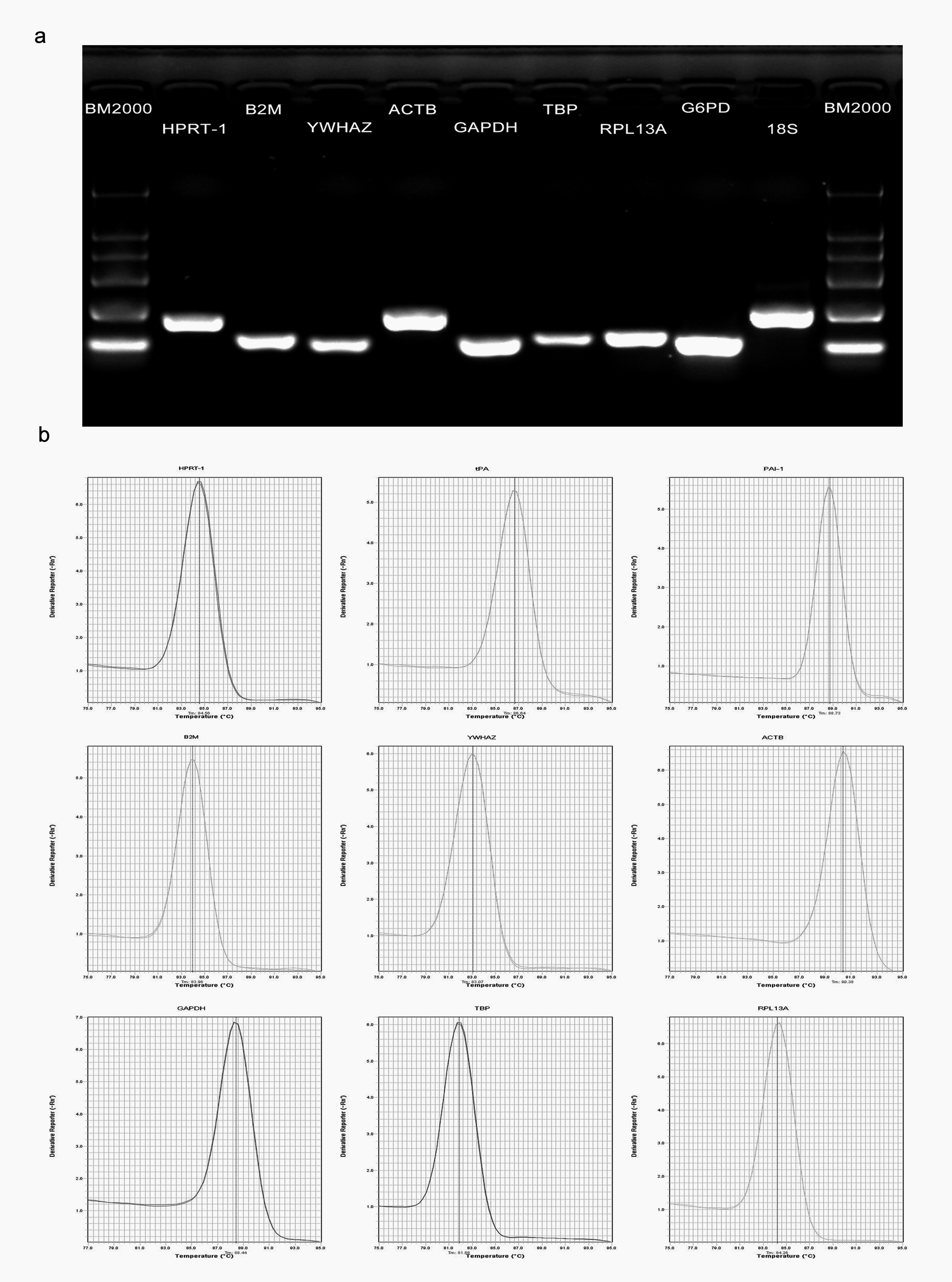

Supplement: Figure S1 — Specificity of qRT-PCR amplification. (a) Amplified fragments were separated by 2% agarose gel. BM2000 represented DNA size marker. The fifth marker band corresponds to 250bp, and the last marker band corresponds to 100bp. (b) Dissociation curves of the nine amplicons showing single peaks. (TIF) [file pone.0067360.s001.tif]

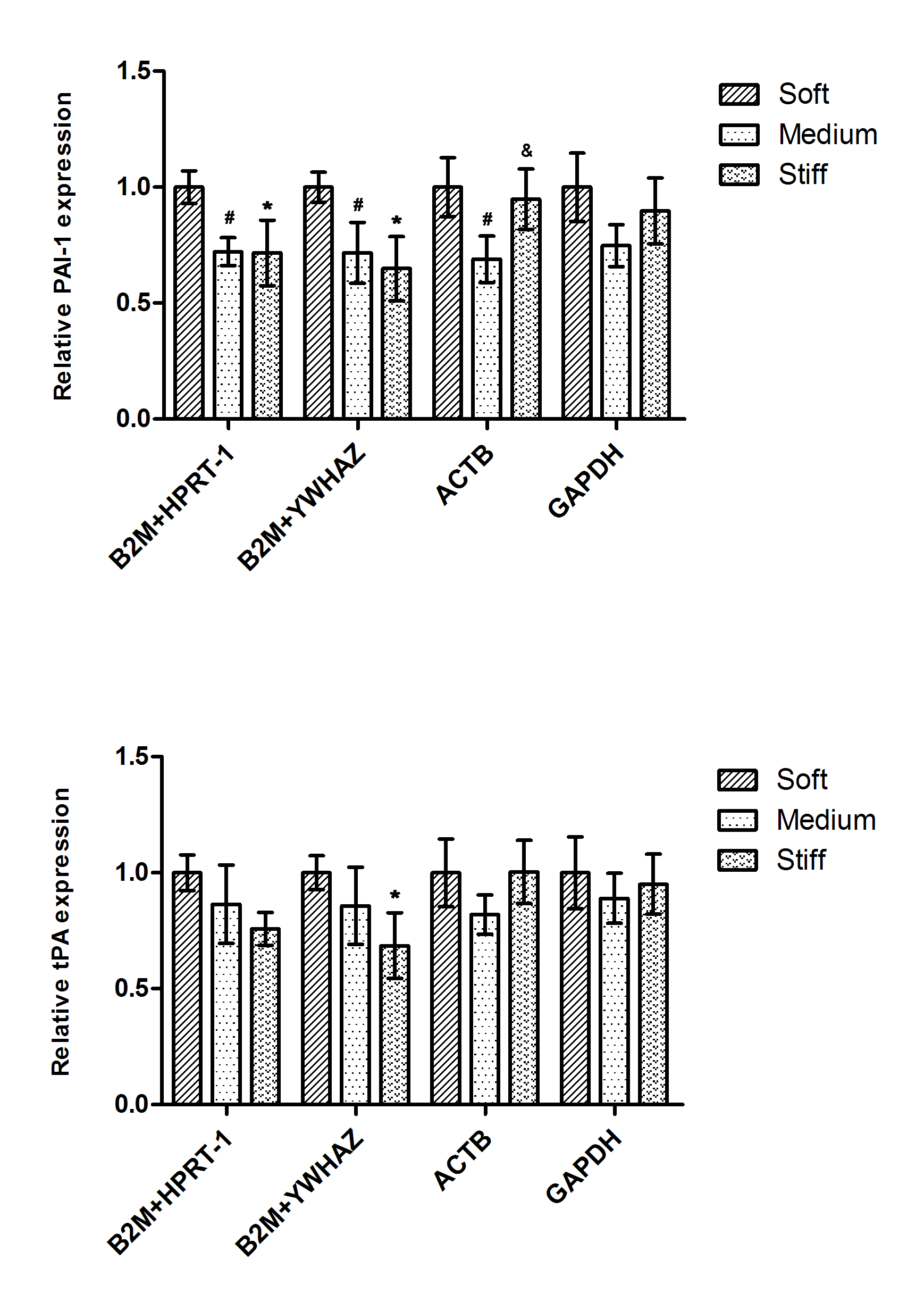

Supplement: Figure S2 — Expression levels of PAI-1 and tPA in endothelial cells on a variety of substrate stiffness. Genes were normalized to individual and/or combined RGs. Results are mean±SD, n = 3; Means were compared by ANOVA followed by Student-Newman-Keuls text. #P<0.05, Medium vs. Soft. *P<0.05, Stiff vs. Soft. &P<0.05, Stiff vs. Medium. (TIF) [file pone.0067360.s002.tif]
