# Supplementary material for: Validation of Reliable Reference Genes for Real-Time PCR in Human Umbilical Vein Endothelial Cells on Substrates with Different Stiffness
Source: PLoS One. 2013 Jun 28;8(6):e67360. doi: 10.1371/journal.pone.0067360 (PMC3696109; doi:10.1371/journal.pone.0067360)
Supplement: Table S1 — Primer Information for YAP, CTGF, tPA, and PAI-1. (DOC) [file pone.0067360.s003.doc]

**Table S1 Primer Information for YAP, CTGF, tPA, and PAI-1**

|  |  |  |  |
| --- | --- | --- | --- |
| **Gene symbol** | **Forward primer** | **Reverse primer** | **Amplicon size (bp)** |
| **YAP** | TTGGGAGATGGCAAAGACAT | CGTTCATCTGGGACAGCAT | 107 |
| **CTGF** | CCCTCGCGGCTTACCG | GGACCAGGCAGTTGGCTCT | 72 |
| **tPA** | GACGTGGGAGTACTGTGATGTG | CCCTCCTTTGATGCGAAACTGA | 85 |
| **PAI-1** | GAGGTGCCTCTCTCTGCCCTCACCAACATT | AGCCTGAAACTGTCTGAACATGTCG | 183 |
